# Tackling the dysregulated immune-checkpoints in classical Hodgkin lymphoma: bidirectional regulations between the microenvironment and Hodgkin/Reed-Sternberg cells

**DOI:** 10.3389/fonc.2023.1203470

**Published:** 2023-05-24

**Authors:** Alessandro Cellini, Federico Scarmozzino, Francesco Angotzi, Edoardo Ruggeri, Angelo Paolo Dei Tos, Livio Trentin, Marco Pizzi, Andrea Visentin

**Affiliations:** ^1^ Hematology and Clinical Immunology Unit, Department of Medicine, University of Padua, Padua, Italy; ^2^ Surgical Pathology and Cytopathology Unit, Department of Medicine, University of Padua, Padua, Italy

**Keywords:** Hodgkin lymphoma, microenvironment, Treg, PD-1, LAG-3, immune checkpoint inhibitors, nivolumab, pembrolizumab

## Abstract

Immune evasion is considered one of the modern hallmarks of cancer and is a key element in the pathogenesis of classical Hodgkin Lymphoma (cHL). This haematological cancer achieves effective avoidance of the host’s immune system by overexpressing the PD-L1 and PD-L2 proteins on the surface of the neoplastic cells. Subversion of the PD-1/PD-L axis, however, is not the sole contributor to immune evasion in cHL, as the microenvironment nurtured by the Hodgkin/Reed-Sternberg cells is a major player in the creation of a biological niche that sustains their survival and hinders immune recognition. In this review, we will discuss the physiology of the PD-1/PD-L axis and how cHL is able to exploit a plethora of different molecular mechanisms to build an immunosuppressive microenvironment and achieve optimal immune evasion. We will then discuss the success obtained by checkpoint inhibitors (CPI) in treating cHL, both as single agents and as part of combination strategies, analysing the rationale for their combination with traditional chemotherapeutic compounds and the proposed mechanisms of resistance to CPI immunotherapy.

## 1 Introduction

From the time of its first description by Thomas Hodgkin to the latest classification of haematological neoplasms, classical Hodgkin Lymphoma (cHL) has always held a peculiar spot amongst haematological cancers with both its clinical and biological characteristics keeping it apart from other forms of lymphoma (1–3). Clinically, cHL is one of the most common cancer types being diagnosed in adolescents, with a characteristic incidence bimodal distribution having peaks in the young adult population and in subjects aged 55 and older. Moreover, it is characterised by good response rates to first-line therapy, with cure rates ranging from 70 to 90% with chemotherapy alone or its combination with radiotherapy, depending on stage and other risk factors, and with second line-therapy allowing for disease eradication in a good portion of relapsed/refractory patients (4).

From a pathological standpoint, cHL is composed of a minority of large mononuclear (Hodgkin) and multinucleated (Reed-Sternberg) neoplastic cells, scattered throughout a rich inflammatory microenvironment (ME) (1). The latter usually constitutes >95% of the tumour mass and consists of mature B and T lymphocytes, plasma cells, eosinophils, macrophages, mast cells, and, to a minor extent, of myeloid suppressor derived cells (MSDCs) and NK cells (5). The relative proportion of such populations and the distribution of each immune cell subset depend on a tight crosstalk between Hodgkin/Reed-Sternberg cells (HRSC) and the inflammatory background, allowing HRSC to shape the immune ME and producing biological niches that sustain their survival and shelter them from immune surveillance.

## 2 The physiopathology of the PD-1/PD-L axis

The overexpression of Programmed Death Ligand 1 (PD-L1) on the surface of HRSC is one of the hallmarks of cHL and is one of the main contributors to its ability to avoid immune-mediated disruption (6, 7).

From a physiological point of view, PD-L1 is a transmembrane protein belonging to the B7 family, usually expressed on the surface of immune cells, professional (such as dendritic cells, macrophages and B-lymphocytes) and nonprofessional (such as epithelial and endothelial cells) antigen-presenting cells (APCs) and many nonhematopoietic cells (8). Alongside PD-L2, PD-L1 exerts its role by binding to the PD-1 receptor expressed on the surface of T-lymphocytes, resulting in an inhibitory signal (9). After the binding of the PD-L proteins to the PD-1 receptor, the immunoreceptor tyrosine-based inhibitory and switch motifs (ITIM and ITSM, respectively) located in the cytosolic end of the receptor are autophosphorylated, allowing for the recruitment of Src homology region 2 containing phosphatase 2 (SHP2), which represents the main effector of the inhibitory signal triggered after the engagement of PD-1 (10–12). After it’s engagement to the cytosolic end of the PD-1 receptor, SHP2 undergoes a conformation change that allows for its activation (13). This phosphatase is then able to inhibit the phosphoinositide-3-kinase (PI3K) and the phospholipase C gamma 1 (PLCG1), interfering with the PI3K/Akt and Ras/MAPK/ERK pathways, which are crucially involved in T-cell activation and their survival after antigen recognition. SHP2 is also able to directly inhibit the function of the leukocyte-specific tyrosine kinase (LCK), which, in turn, is responsible for the phosphorylation of fundamental proteins involved in T-cell activity such as ZAP70, CD3ζ and PKCθ (14, 15). Moreover, an activated PD-1 is able to interfere with the activity of the casein kinase 2 (CK2) by reducing both its activity and its mRNA levels. This, in turn, allows for continued phosphatase activity by the phosphatase and tensin homolog (PTEN) protein, which is inhibited by CK2-mediated phosphorylation during T-cell activation, further interfering with the PI3K/Akt pathway and thus truncating the TCR downstream signals (16).

**Figure 1:**
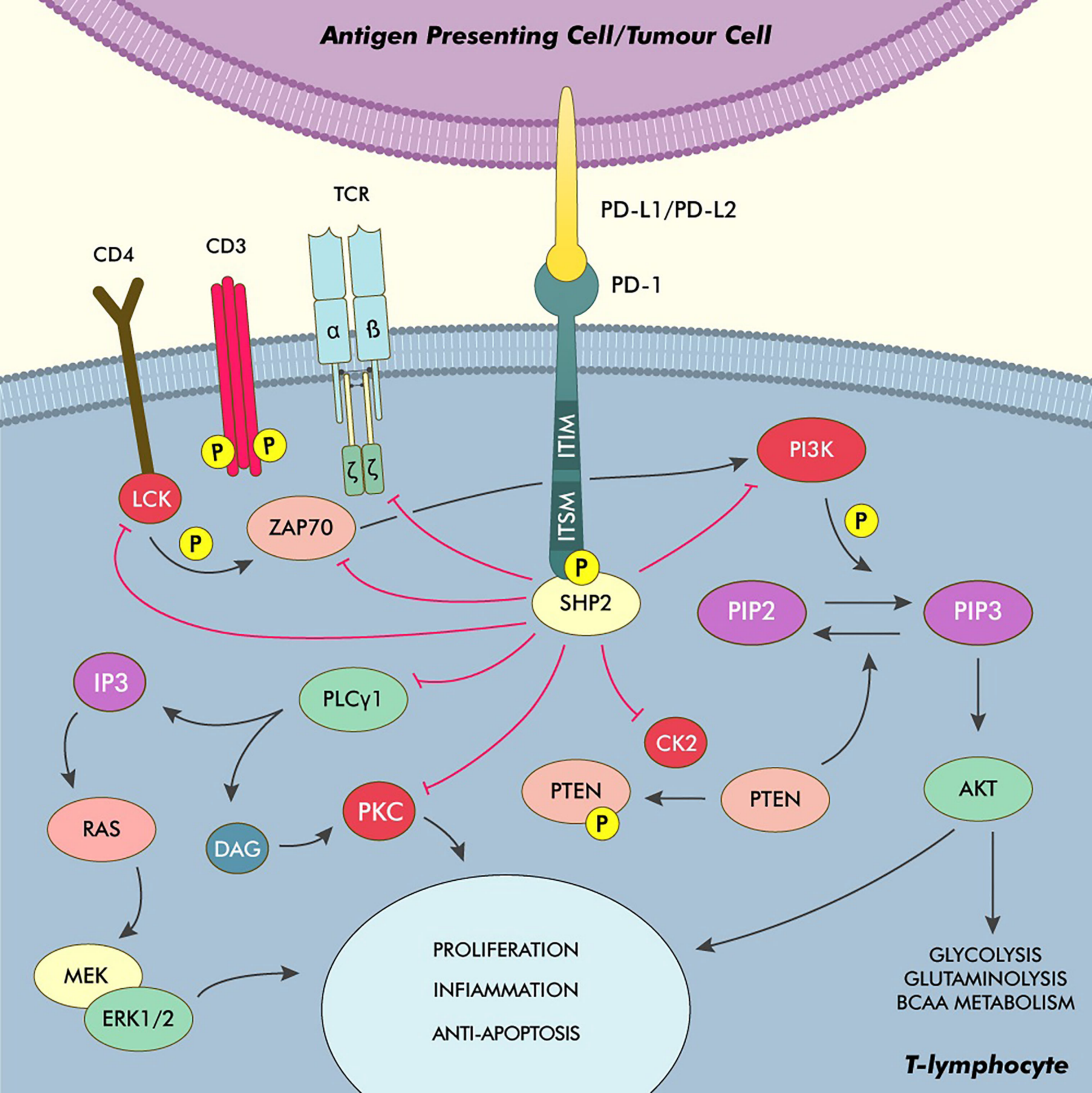
Phyisiology of the PD-1/PD-L1 axis and main pathways of immune modulation in cHL. After binding to PD-L1/PD-L2, PD-1 undergoes autophosphorylation on its ITSM and ITIM, recruiting and activating SHP2. This phosphatase acts as the main effector, truncating the TCR activation signals by inhibiting ZAP70, LCK and the TCRζ subunit. The inhibition of the PI3K/Akt pathway also stops the T-cell from switching its metabolism from fatty acid oxidation to glycolysis, glutaminolysis and the utilisation of BCAA, which is a core element of T-cell activation. SHP2 also inhibits the PI3K/Akt pathway by directly interfering with the PI3K activity and by inhibiting the kinase CK2, thus allowing PTEN to dephosphorylate PIP3. Moreover, SHP2 directly inhibits PLCγ1 thus hindering the production of IP3 and DAG and the activation of PKC and the Ras/MEK/ERK pathway. DAG, Diacylglycerol; IP3, Inositol triphosphate; PKC, Protein kinase C.

Furthermore, the inhibition of the PI3K/Akt pathway through the activation of the PD-1/PD-L1 axis induces metabolic changes, making in T-cells unable to exploit glycolysis, glutaminolysis and branched-chain amino acid metabolism for energy production, all of which are key aspects of the activated T-cell phenotype. Moreover, PD-1 derived signals induce an increase in fatty-acid oxidation through upregulation of key enzymes involved in lipid metabolism, a metabolic switch associated with cell longevity capability of reacting to antigenic stimuli (**Figure 1**) (17).

## 3 Immune evasion in Hodgkin’s Lymphoma

As stated before, the PD-1/PD-L axis plays a pivotal role in cHL’s ability to avoid immune recognition. One of the most characteristic genetic lesions, which is found in almost all cases of cHL, is the amplification of the 9p24.1 chromosomal region, containing the *CD274* and the *PDCD1LG2* genes, encoding, respectively, for the PD-L1 and PD-L2 proteins. Overexpression of these genes directly translates into a higher expression of both proteins on the surface of HRSC, and, consequently, direct inhibition of the T cells in the surrounding tumour ME. Moreover, the same genomic region includes the *JAK2* gene, and its amplification leads to an overactivation of the JAK/STAT pathway, which, in turn, acts as a positive transcriptional regulator to the genes encoding for PD-L1 and PD-L2, further enhancing the HRSC ability to pathologically express these antigens on their surface (6, 7, 18).

Epstein-Barr Virus (EBV) positive cHL is rarely characterised by the 9p24.1 amplification, and, as such, is not able to exploit the genetic pathways detailed above to manipulate the PD-1/PD-L immune checkpoint. The latent membrane viral protein 1 (LMP1), produced as a consequence of EBV infection, is however able to increase transcription levels of the *CD274* and *PDCD1LG2* genes through stimulation of the AP-1 and JAK/STAT signalling pathways, therefore making EBV-infected HRS cells as proficient in immune evasion as their EBV-negative counterparts (19).

**Figure 2:**
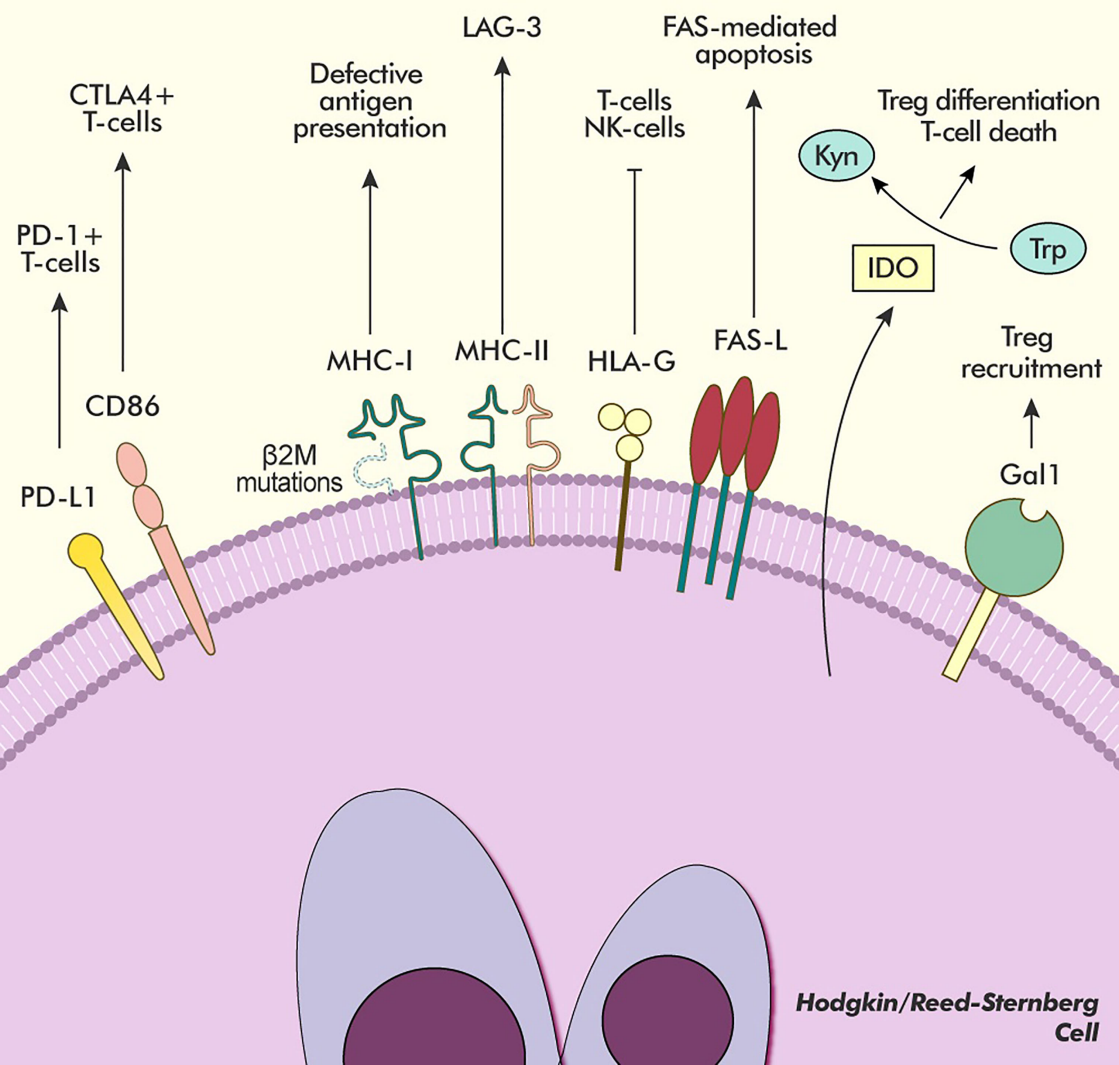
Main pathways of immune modulation in cHL. HRSC avoid immune detection with several different mechanisms. The overexpression of PD-L1 and CD86 allows for interaction and inhibition with two different subsets of T-lymphocytes (PD-1+ and PD-1-/CTLA-4+, respectively). Frequent mutations in the β2M gene hinder antigen presentation to T-cells by producing a defective MHC-I receptor, whose interactions are replaced by the inhibitory HLA-G receptor. Moreover, the MHC-II receptor expressed by HRSC is able to bind the inhibitory LAG-3 receptor expressed on exhausted T-cells in the microenvironment further downregulating their activity. The overexpression of FAS-L induces apoptosis in lymphocytes bearing the FAS receptor. The presence of the IDO enzyme, produced both by HRSC and microenvironment cells, causes Trp depletion, which forces a regulatory differentiation of T-cells, and Kynurenine accumulation, which induces T-cell death. The expression of Gal1 on the surface of HRSC further increases the recruitment of Treg to the microenvironment. Kyn, Kynurenine; Trp, Tryptophan.

Pathological overexpression and dis-regulation of the PD-L1 and PD-L2 surface proteins, however, are not the only mechanisms that define cHL’s ability to manipulate the host’s immune system. Having a B-cell origin (20), HRSC derive from APCs and, as such, are expected to express both class I and class II Major Histocompatibility Complexes (MHC-I and MHC-II, respectively). More than half of cHL cases are characterised by the loss of surface expression of MHC-I due to mutations in the gene encoding for Beta-2 microglobulin (β2M), thus impinging antigen presentation to CD8+ T-cells (21, 22). The loss of MHC-I on HRSC is also associated with overexpression of HLA-G, a noncanonical MHC-I receptor that is able to bind to the inhibitory ILT2 and ILT4 surface proteins on other immune effector cells, as well as to CD8 on the surface of T and NK cells, triggering FAS-mediated apoptosis, a process also stimulated by the strong expression of FASL on HRSC (**Figure 2**) (22, 23).

**Figure 3:**
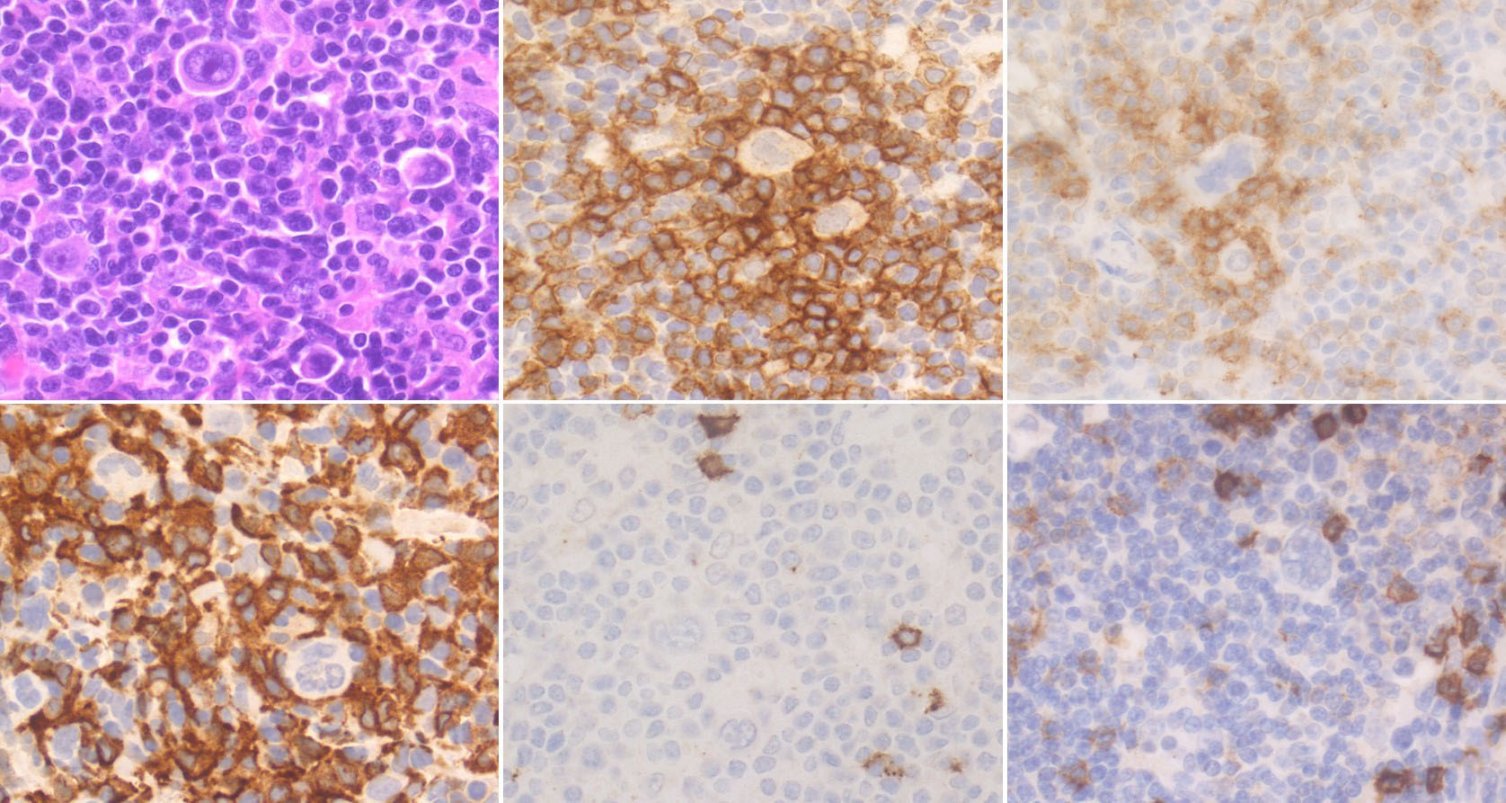
Histological and Immunohistochemical features of cHL’s microenvironment. Scattered HRSCs are embedded in a reactive microenvironment (ME). CD4+ PD-1+ lymphocytes are enriched around HRSCs, often organised in rosettes. Numerous CD163+ M2 macrophages are also present, while CD8+ T lymphocytes and CD20+ B lymphocytes are sparse. (H&E and immunoperoxidase stains; original magnification 63x).

The nurturing of an immune-suppressive biological niche, whose members are directly involved in dynamic interactions among themselves and with HRSC throughout cell-to-cell contacts and soluble factors, is another key element in the biology of cHL (24). Through the release of many cytokines and chemokines, HRSC recruit circulating immune cells, reshaping their functions and promoting their local expansion and spatial distribution (**Figure 3**). For these processes, the most relevant factors are IL5, IL7, IL8, IL9, CCL5, CCL17, CCL20 and CCL22, which have been variably associated with recruitment/activation of eosinophils, neutrophils, B and T lymphocytes, mast cells and specific macrophage subsets (25, 26). Beside these soluble factors, many surface receptors on HRSCs promote their growth/survival, through direct interaction with immune cell subsets and/or through binding of ME-derived cytokines. These include the activating receptors CD30 and CD40, and interleukin/chemokine receptors, such as IL7R, IL9R, IL13R, TACI and CCR5 (27–30). The engagement of such receptors activates downstream signalling pathways, which are pivotal for the pathogenesis of cHL. These include the PI3K/AKT pathway (interaction between CD30 and CD30-ligand on mast cells and eosinophils), the NF-κB pathway (interaction between CD40 and CD40-ligand on CD4+ T cells), and the JAK/STAT pathway (interaction of HRSC surface receptors with several ME-derived cytokines) (31–39).

Besides providing survival and proliferation stimuli, tumour ME is able to directly interfere with the host’s immune recognition of HRSC. In detail, the presence of regulatory T cells (Treg) among the inflammatory infiltrate exerts a major pathogenic role by secreting large amounts of immune inhibitory IL10 (40, 41). In cHL, Treg are recruited by chemoattractants of HRSC derivation, such as CCL17/TARC, CCL22, CCL5, IL-4, IL-5, IL-10 and IL-13. Treg infiltration is also facilitated by the over-expression of galectin-1, a beta-galactoside-binding protein that mediates cell-to-cell and cell-to-matrix interactions (42). Other mechanisms involved in Treg enrichment include HRSC-induced trans-differentiation of bystander T helper cells (43, 44) and Treg recruitment by cHL-associated accessory and myeloid cells. This is specifically mediated by M2 macrophages, mast cells and MDSCs. Moreover, the expression of the indoleamine 2,3-dioxygenase (IDO) enzyme by both HRSC and other elements of the tumour ME allows for the degradation of Tryptophan in the cHL biological niche, suppressing the activity of NK cells and forcing T cells to acquire a Treg phenotype (45). In addition, Kynurenine, which is the main Tryptophan metabolite, is able to inhibit antigen recognition-derived proliferation and to induce T-cell death (46, 47).

The lymphocyte infiltrate is also rich in cells expressing high levels of lymphocyte-activation gene 3 (LAG-3), which, alongside PD-1 and TIM3, defines a subset of T-cells that have undergone activation and subsequent exhaustion due to chronic antigen stimulation. LAG3 is another contributor to the mainly tolerogenic attitude exerted by T cells in cHL due to its ability to bind MHC-II with greater affinity than CD4 leading to the inhibition of TCR signalling, proliferation and cytokine secretion by antigen specific T-cells (48–50). Interestingly, a second T-cell population that expresses CTLA-4 but is PD-1 and LAG-3 negative has been recently described to be in close contact with HRSC, together with the identification of strong expression of CD86 on the latter cell type, suggesting that cHL is able to exploit yet another known immune checkpoint to enhance its survival with limited crosstalk between the CD86/CTLA-4 and PD-1/PD-L1 axes, as they appear to engage on different T-cell populations (**Figure 2**) (51).

Tumour infiltrating macrophages are also another key actor in the immunosuppressive inflammatory infiltrate that characterises cHL (52). These cells, under the stimulation of IFN-gamma produced in the surrounding environment, express high levels of PD-L1, and as such, they participate in the suppression of T-cell response both directly, by binding PD-1 on the surface of T-lymphocytes, and indirectly, by impeding the interaction between T cells and HRSC (53). Of note, the high levels of PD-L1 seen in cHL-associated macrophages may be partially due to trogocytosis of PD-L1-loaded HRSC membrane patches (54).

M2 macrophages are directly induced by HRSCs through TNF, IL-10, TGF-β, GM-CSF, IL-13 and CCR5 secretion (55, 56). Mast cells are attracted by HRSC-derived IL13 and promote Treg skewing *via* TGF-β. This growth factor also stimulates stromal remodelling by fibroblast proliferation and collagen production (57). Finally, MDSCs support immune suppression *via* TGF-β, IL-10 and peroxynitrite production and *via* arginase-mediated depletion of ME arginine, an immune-metabolite responsible of T cell proliferation and activation (58–62).

## 4 Checkpoint inhibitors in clinical trials

### 4.1 Pivotal trials

Given the critical role played by the PD-1/PD-L axis in cHL’s avoidance of physiological immune surveillance, and its overexpression of PD-L1 on the surface of the majority of HRSC, this specific immune checkpoint became a prime candidate for targeted therapy after the success obtained by checkpoint inhibitors (CPI) in treating solid malignancies such as melanoma (63, 64) or non-small-cell lung cancer (65–67).

**Figure 4:**
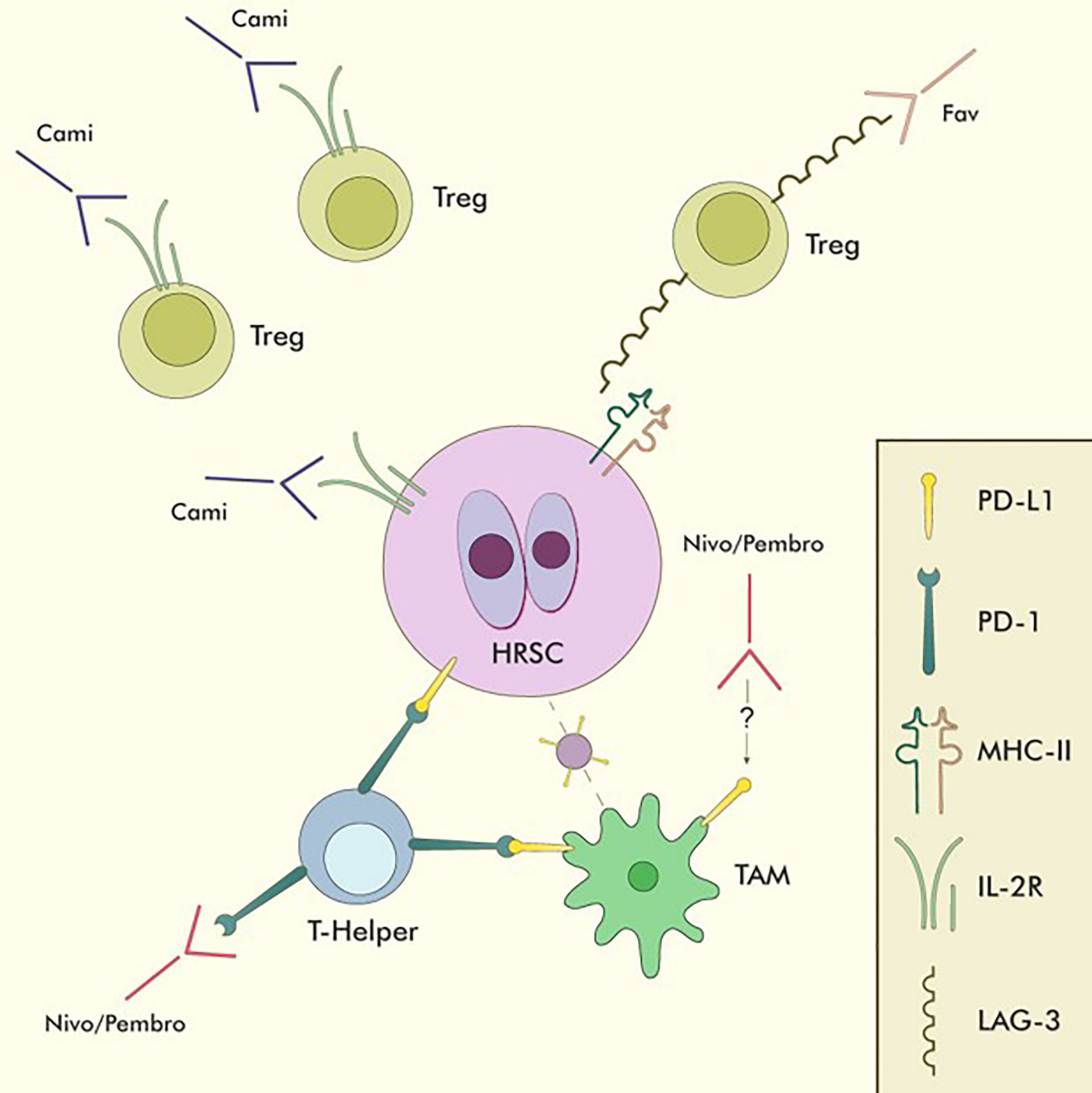
Immune-checkpoint inhibitors in cHL. Nivo and Pembro are the only approved CPIs for cHL. They act mainly by binding PD-1+ T-helper lymphocytes in the tumour microenvironment, thus reducing the inhibitory effect of PD-L1 overexpression on HRSC and restoring T-cell mediated anti-tumour immunity. They also reduce the interactions between PD-1+ T-helper lymphocytes and PD-L1+ TAMs, allowing T-helper lymphocytes to closely interact with HRSC without the inhibitory influence of TAMs. Moreover, Nivo has been shown to be able to directly reduce PD-L1 expression on TAMs through mechanisms which are still being investigated. Favelizumab (Fav) is a LAG-3 directed monoclonal antibody currently being tested in combination with anti-PD-1 CPIs within clinical trials, acting as an inhibitor of the LAG-3/MHC-II interaction, thus impinging Treg function and freeing up MHC-II molecules for CD4/MHC-II interactions on activated T-helper lymphocytes. Camidanlumab-Teserine (Cami) is a CD25 directed drug-antibody conjugate that acts by directly binding to IL-2Rα (CD25) on the surface of HRSC and delivering its toxic payload directly to neoplastic cells. As CD25 is also expressed on the surface of Tregs, the activity of Cami might be partially due to Treg depletion and subsequent restoration of competent anti-tumour immunity. TAM, Tumour-associated macrophage.

Nivolumab (Nivo) and Pembrolizumab (Pembro) are two PD-1 directed monoclonal antibodies that recognise their antigen on the surface of T-lymphocytes, thus interfering with their interaction with neoplastic cells and restoring their ability to react against the anomalous cancer antigens (**Figure 4**).

Nivo was first approved in 2016 for relapsed/refractory (R/R) cHL based on the results of the CheckMate-205 trial, which enrolled 243 patients with R/R cHL after failure of high dose chemotherapy and autologous stem cell transplantation (ASCT). Patients were assigned to 3 cohorts according to their prior exposure to Brentuximab Vedotin (BV) and received Nivolumab at a dose of 3 mg/kg until progression, unacceptable toxicity or consent withdrawal. In this heavily pretreated patient population (4 median lines of previous therapy), an overall response rate (ORR) of 72% was documented, with a complete remission rate (CRR) of 33% and a median time to first objective response of 2.1 months (across cohorts A, B and C). After a median follow-up time of 18 months, the median progression-free survival (PFS) was 14.7 months, while the median overall survival (OS) was not reached, and the 1-year OS was 92%. In patients who obtained a complete response (CR) median duration of response was 20.3 months, with a median PFS of 22.2 months and a 12-month survival rate of 100% (68, 69).

On the other hand, Pembro was first tested in haematological malignancies in the KEYNOTE-013 trial, which included a small cohort of R/R cHL patients (n=31) where it yielded promising results. Its activity in cHL was subsequently more formally tested in the KEYNOTE-087 trial, which led to its approval in 2017 in R/R cHL patients not eligible for ASCT. This trial was specifically designed for cHL and enrolled a total of 210 patients with a median of 4 previous lines of therapy, treating them with a fixed dose of 200 mg of Pembro every 3 weeks for up to 2 years, or until disease progression, unacceptable toxicity or consent withdrawal. In this setting, Pembro achieved an ORR of 71.0% with a 27.6% of CRR. The median DOR was 16.6 months. In its most recent follow-up report (median follow-up of 62.9 months) median PFS was 13.7 months and median OS was not reached. In patients who achieved CR, median DOR was not reached, with 4 patients obtaining a response lasting for ≥60 months, while median PFS was 56.5 months, and median OS was not reached. The 5-year PFS and OS rates for patients achieving CR were 44.3% and 82.8%, respectively. These results are similar to those observed within the small cohort of cHL patients in the KEYNOTE-013 trial (70–73).

In the setting of R/R cHL patients who progressed after ASCT or were not eligible for the transplant procedure, the activity of Pembro was directly compared against that of BV in the phase 3 randomised KEYNOTE-204 trial, of which an interim analysis was recently reported. The study enrolled 304 patients (151 assigned to the Pembro arm and 153 to the BV arm). Among these, 37% had already undergone ASCT, 44% were defined as having chemorefractory disease and 18% had undergone a single line of previous therapy. ORR by investigator review were 68.2% in the Pembro treatment group compared to 50% in the BV treatment group, with a CRR of 26% and 24%, respectively. After a median follow-up time of 25.7 months, the superiority of Pembro compared to BV in this setting was established, with a median PFS of 13.2 months and 8.3 months (HR 0.65, p=0.0027), respectively. Overall survival estimates were not intended to be part of the interim analysis reported, and as such were not described. Median time to objective response was similar among the two groups (2.8 months), with a median duration of response of 20.7 months (95% CI 12.4 to not reached) in the Pembro cohort and of 13.8 months (95% CI 5.8 to not reached) in the BV cohort. Interestingly, the subgroup analysis reported in this interim report showed a particularly favourable efficacy profile of Pembro in the primary refractory patients, where median PFS was 12.5 months and thus comparable to the one depicted for the whole patient population. BV, on the other hand, achieved a median PFS of only 5.5 months in primary refractory patients (74).

These trials depict the effectiveness of CPI therapy in a highly pretreated cHL patient population, including multiple relapsed and chemorefractory patients, who have been historically very challenging to treat, obtaining a response in around 70% of the treated individuals. As satisfactory as this might be, however, only one third of treated patients manage to achieve CR, and very few obtain long lasting disease control, as shown by a PFS ranging from 12 to 14 months across the 3 pivotal studies. At this moment in time, the optimal approach to patients who do not obtain complete remission with CPIs or whose disease becomes resistant to these immunomodulating agents is still being debated upon, as is the optimal approach to consolidating the obtainment of a complete response.

As CPIs were first approved in non-haematological malignancies, the need to treat patients who progressed during CPI immunotherapy, or that were refractory to it, first arose in solid oncology, and a trend towards a better response to the subsequent chemotherapy regimens after exposure to CPIs was observed (75, 76). On the back of this evidence, a few reports on the effectiveness of chemotherapy after CPI treatment in cHL have been published in recent times. Although limited by their retrospective nature, the relatively small number of patients enrolled and the quite diverse array of post-CPI therapies, these studies report an ORR ranging from 59% to 93% (CRR ranging from 42% to 100%) in such heavily pretreated patient populations (median number of therapies were ≥4 in all studies reported), with most of the patients being refractory to the last chemotherapeutic agent administered before CPI therapy, and some patients responding to cytotoxic agents that they had proven to be refractory to prior to CPI exposure (77–80).

These results make us glimpse at the ability of CPIs to not only direct the host’s immune response against the tumour, but also to restore its sensitivity to classical chemotherapeutical compounds with molecular mechanisms that are still widely unknown. Of note, one among these retrospective studies depicts a particularly high response rate in patients undergoing chemotherapy with the Gemcitabine-based regimen, and the authors speculate that these high response rates might be attributed to the ability of Gemcitabine to influence the tumour-directed immune response (79).

Moreover, evidence obtained from the pivotal Pembro and Nivo trials, as well as real-life studies, even in the event of a complete response to CPIs, long term disease control is not assured, whereas consolidation with ASCT or allogeneic stem cell transplant (alloHSCT) might represent a potentially curative approach. A recent large retrospective analysis reported on the effectiveness of alloHSCT after PD-1 directed therapy in cHL. With a median follow-up of 24 months, the 2-year graft and relapse free survival, PFS and OS were 47%, 69% and 82%, respectively, comparing favourably against historical data obtained in non-anti-PD1 exposed similar patient populations. Past experiences with alloHSCT after exposure to CPIs led to some safety concerns for higher risk of graft-versus-host disease (GvHD). Interestingly, the analysis reported here further confirmed a higher risk of GvHD, that was however much milder in patients with a time interval of >80 days between the last CPI dose and the transplant procedure. Thus, anti-PD-1 monoclonal antibodies can increase the reactivity of the allogenic cellular products, which in turn translates both into a higher risk of GvHD and a higher graft-versus-lymphoma effect, justifying the higher PFS seen in this study, and the lower incidence of relapse in patients undergoing haploidentical alloHSCT. Interestingly, the usage of post-transplant Cyclophosphamide was able to reduce the GvHD risk without impacting on the ability of the allogeneic stem cell product to obtain disease control (81).

### 4.2 Combination therapies

Given the good results achieved by PD-1 targeted CPI monotherapy in the heavily pretreated patient populations described above, an effort is being made to bring these agents further up in the cHL’s treatment algorithm.

In this framework both Nivo and Pembro are being tested as part of combination strategies both with other targeted therapies and with classical chemotherapeutic compounds. While the former therapeutic regimen would have the advantage of a chemo-free approach to cHL, the latter exploits the ability of some classical chemotherapeutic agents to modulate the host’s immune response against cancer, as well as the direct sensibility of HRSC to their cytotoxic properties. This is the case with drugs such as Gemcitabine, Cisplatin and Doxorubicin, which have been part of the therapeutic armamentarium against cHL for quite some time. In particular, Gemcitabine has been found to increase MHC-I expression on the surface of cancer cells by activating cellular response pathways that influence the levels of β2-microglobulin. Moreover, Gemcitabine is able to modify the structure of the proteasome machinery, increasing their antigenic repertoire, thus markedly increasing the neoplastic cell immunogenicity (82). This nucleoside analogue is also able to reduce the percentage of myeloid-derived suppressor cells in the tumour ME and to cause Treg depletion thus further enabling an effective anti-tumour immune response, at least in the preclinical setting (83–85). Furthermore, Doxorubicin, like Cisplatin, is able to induce the so-called immunogenic cell death through the phosphorylation of eukaryotic translation initiation factor 2 subunit alpha (eIF2α), a mechanism that is independent from the inhibition of the Topoisomerase-II enzyme, which is one of the main targets of these antineoplastic agents (86, 87). The phosphorylation of eIF2α is an integral part of a stress response mechanism that ultimately leads to the activation of caspase-8 dependant apoptosis and the exposure of Calreticulin on the cellular surface (88). This kind of cellular death is also accompanied by the release of ATP and cancer nucleic acids in the extracellular matrix, as well as by the exposure of “eat-me” signals other than Calreticulin on the surface of the neoplastic cells. This, in turn, translates into an increased uptake of fragments of neoplastic cells by dendritic cells, which enhances antigen presentation and contributes to the development of a directed immunological response (89–91).

The results from two phase 2 trials exploring the combination of Nivo and Pembro with standard chemotherapeutic regimens have recently been reported. In the first one, a PET-guided sequential approach containing Nivolumab and the Ifosfamide-Carboplatin-Etoposide (ICE) chemotherapeutic protocol was utilised. Patients were to receive up to 12 cycles of Nivo, and, in the event of progression, stable disease (SD) or partial response (PR) they would then proceed to 6 cycles of Nivo-ICE (NICE) therapy. Patients in CR or PR at the end of the therapy protocol, or in CR after only 6 cycles of Nivo, would then proceed to stem cell mobilisation and ASCT. The trial population was composed of 43 R/R cHL patients, of which 44% had primary refractory cHL. The ORR and CRR after 12 Nivo cycles were 81% and 71%, respectively, and went up to 93% and 91% after the completion of the protocol therapy. Nine patients went on to the NICE combination therapy (of which 3 shifted from Nivo to NICE after 3 cycles of Nivo due to PD/SD), all achieving at least a PR, with a CRR of 89%. With a median follow-up time of 30.7 months, the 2-year PFS and OS estimates were 72% and 95%, respectively (92). Within the same trial, a second cohort (*n=*35) explored a non-PET-guided approach in high risk patients, where a single cycle of Nivo would be directly followed by 2 or 3 cycles of NICE. Preliminary results of this cohort have been recently presented at the 2022 ASH Congress, where an ORR and a CRR of 100% and 88% were reported (93).

The second phase 2 trial exploring PD-1 blockade with classical chemotherapy tested the combination of Pembro with Gemcitabine, Vinorelbine and Doxil (GVD). In this study, patients underwent up to 4 cycles of Pembro-GVD, followed by consolidation with high-dose chemotherapy and ASCT. Patients in CR after 2 cycles of Pembro-GVD directly proceeded to ASCT consolidation. Thirty-nine patients were registered in the trial, and 41% of them had primary refractory disease. Among the 38 eligible for response assessment, reported ORR and CRR were 100% and 92% after 2 cycles of Pembro-GVD, respectively. Eight patients went on to receive two additional cycles of the protocol therapy, and at the end of salvage therapy ORR and CRR were 100% and 95%. Thirty-six patients subsequently proceeded to ASCT, and, with a median follow-up of 13.5 months, all of them remain alive and progression-free. Of note, engraftment syndrome (defined as a high-grade non-infectious fever in the protocol) was reported in 68% of the patients who underwent the transplant procedure. All cases were treated with corticosteroids and completely resolved. Interestingly, there was no association between engraftment syndrome and previous immune-related adverse events (94).

In both of the studies exploring the combination of Pembro/Nivo and classical chemotherapy, a favourable toxicity profile was noted, with adverse events consistent with the ones reported with the usage of anti-PD-1 antibodies in monotherapy and the specific chemotherapeutic agents utilised in the two trials.

As stated above, combination therapies involving both PD-1 directed CPIs and other targeted agents are also being explored. A phase 1-2 study explored the BV-Nivo combination as first salvage therapy in R/R cHL patients candidable to ASCT. This trial enrolled 93 patients, with 42% of subjects having primary refractory disease, achieving an ORR of 85%, with a CRR of 67%. After a median follow-up time of 34.3 months, median OS and PFS were not reached, and the estimated 3-year PFS and OS rates were 77% and 93%, with 74% of patients being able to undergo ASCT directly after the planned BV-Nivolumab cycles. Of note, the 2-agent combination did not have a significant impact on peripheral blood stem cell collection, and had a favourable toxicity profile, and no treatment-emergent adverse effects were registered other than the ones already experienced with each drug when given as monotherapy (95). A summary of trials utilising CPIs in R/R cHL is available in **Table 1**.

**Table 1:** Summary of clinical trials exploring the efficacy of Nivolumab and Pembrolizumab in R/R cHL.

Trial Name	Treatment regimen	Treatment setting	Patient number	Last reported median follow up (range)	ORR (CRR)	Median duration of response (95% CI)	Median PFS (95% CI)
CheckMate-205 (NCT02181738) (69)	Nivolumab 3 mg/kg q2w	R/R cHL (median of 4 previous lines of therapy)	243	18 months	72.0% (33.0%)	16.6 months (12.8 – NR)	14.7 months (11.3 – 18.5)
KEYNOTE-013 (NCT01953692) (70, 71)	Pembrolizumab 10 mg/kg q2w	R/R cHL (median of 5 previous lines of therapy)	31	52.8 months	58.0% (19.0%)	NR (3.7 months – NR)	11.4 months (4.9 – 27.8)
KEYNOTE-087 (NCT02453594) (72, 73)	Pembrolizumab 200 mg q2w	R/R cHL (median of 4 previous lines of therapy)	210	62.9 months	71.0% (27.6%)	16.6 months (11.8 – 27.1)	13.7 months (11.1 – 19.4)
KEYNOTE-204 (NCT02684292) (74)	Cohort A: Pembrolizumab 200 mg q3w Cohort B: BV 1.8 mg/kg q3w	R/R cHL (82% of patients in both cohorts having ≥2 previous lines of therapy)	304 (A: 151 B: 153)	25.7 months	A: 68.2% (26.0%) B: 60.1% (24.0%)	A: 20.7 months (12.4 – NR) B: 13.8 months (5.8 – NR)	A: 13.2 months (10.9 – 19.4) B: 8.3 months (5.7 – 8.8)
NICE (Cohort A) (NCT03016871) (92)	Response adapted Nivolumab 240 mg q2w – ICE	Second line of therapy	43	30.7 months	88% (62%) after 3 cycles of Nivolumab 91% (88%) at the end of protocol	NR	NR 2-year PFS 72% (58 – 63)
NICE (Cohort B) (NCT03016871) (93)	Nivolumab 240 mg q2w – ICE	Second line of therapy, high risk patients	35	12.8 months (0.3 – 25.6)	100% (88%)	NR	NR 1-year PFS 90% (66-98)
Pembro-GVD (NCT03618550) (94)	Pembrolizumab 200 mg – GVD q3w	Second line of therapy	39	13.5 months (2.7 – 27.1)	100% (95%)	NR	NR 1-year PFS 100% (NR – NR)

CRR, Complete response rate; GVD, Gemcitabine-Vinorelbine-Liposomal Doxorubicin; ICE, Ifosfamide-Carboplatin-Etoposide; NR, Not reached; ORR, Overall response rate; OS, Overall survival; PFS, Progression-free survival.

CPI-based therapy has also been explored in combination with AVD in the front line setting of cHL (96, 97). More recent approaches to individualise anti–PD-1-based first-line cHL treatment by on-treatment risk stratification include the GHSG INDIE (NCT04837859). To our knowledge, INDIE will be the first trial investigating a chemotherapy- and radiotherapy-free first line cHL treatment in optimally responding patients. Such individualised immunotherapy will potentially further enhance the benefits derived from anti–PD-1 in cHL by reducing or even avoiding exposure to chemotherapy and/or radiotherapy.

## 5 Resistance to checkpoint inhibition in Hodgkin lymphoma

All the evidence discussed above demonstrates how a large percentage of cHL patients can benefit from CPI-based therapy in various phases of their disease history. The relatively high ORR seen with Nivo and Pembro monotherapy, obtained mainly in populations of patients that are canonically considered harder to treat, shows how this cancer can be intrinsically sensible to such therapies. However, the presence of non-responding patients, as well as the limited duration of responses hints at the existence of resistance mechanisms to checkpoint inhibition that can be present at baseline, in the first case, or that can be acquired during therapy, in the second one.

The mechanisms of CPI therapy resistance in cHL are, at this point in time, far from being completely understood. However, the utilisation of such drugs in the treatment of other malignancies predates their use in cHL, and, as such, evidence on how these solid tumours resist immune checkpoint blockade is becoming clearer and can help us glimpse into cHL’s own resistance mechanisms to checkpoint inhibition. The description of these mechanisms, unproven to directly take part in primary or acquired resistance to CPIs in the specific setting of cHL, is beyond the scope of this review and has been done elsewhere (98, 99). There are, however, some resistance mechanisms that once again set cHL apart from other malignancies and are worth noting.

When anti-PD1 monoclonal antibodies were first developed, the physiopathological basis that was supposed to be behind their efficacy was that the removal of the inhibitory signal coming from the PD-1/PD-L1 axis would rescue CD8+ T cells from their exhausted state, eliciting a direct killer response against the tumour. This has been demonstrated to be the case in different solid tumours (100), and, coherently, the loss of expression of MHC-I molecules on the surface of neoplastic cells is one of the described CPI resistance mechanisms (98, 99). As described above, the loss of MHC-I due to β2M inactivating mutations can be observed in roughly 70% of the cases of cHL. However, the high response rates to CPIs, points to the fact that the activity of these drug in cHL is not based on the direct release of killer T-cell inhibition but rather on the recognition of cancer epitopes exposed on MHC-II molecules by CD4+ T-cells. Indeed, the inflammatory ME appears to be enriched for Th1 polarised CD4+ T-cells, who express an effector memory phenotype (101), characterised, among other peculiarities, by a medium level of expression of PD-1 and a peculiar sensibility to PD-1 targeted therapeutic approaches (**Figure 3**) (102). Coherently, the lack of expression of MHC-I was not associated with reduced CPI effectiveness in a recent report, whereas MHC-II expression was positively associated with better PFS (103). Another proposed mechanism of resistance to checkpoint inhibition is the reduced tumour mutational burden (TMB) seen in some cHL cases (104, 105). As high TMB was one of the first biomarkers associated with a high probability of response to CPI in solid tumours due to the higher production of neoantigens within the neoplastic cells, a low TMB in cHL, combined with the defective antigen presentation associated with the lack of MHC-I expression, might explain some cases of primary resistance to CPIs. In this context, combination therapy with chemotherapeutic drugs that can modify the tumour’s antigenic repertoire might become particularly appealing. The proposed role played by CD4+ T-cells in the response to CPIs seen in cHL has been recently challenged by the analysis of selected patients enrolled in the NIVAHL trial, for which tissue samples obtained at diagnosis and after the first days of Nivo monotherapy were directly compared. Herein, the authors report the complete disappearance of HRSC in up to 50% of the biopsies analysed after the start of therapy, showing no enrichment for T lymphocytes in the inflammatory infiltrate, but rather depicting a reduction of CD4+, LAG-3+ regulatory Th1 polarised T-cells, as well as of PD-1+ tumour associated macrophages. The clinical and pathological responses documented in this study were not significantly associated with MHC-I or MHC-II expression, as well as to clonal T-cell expansion or the increase in T-cell cytotoxic response (106).

While mechanisms of primary and secondary resistance to CPIs in cHL are still being formally assessed, it is tempting to speculate that the peculiar biological characteristics of HRSC and their ME described elsewhere in this paper might play a big role in the loss of an achieved response or the lack thereof seen in some cases. The overexpression of IDO and LAG-3 on T cells and other accompanying inflammatory cells, or the expression of molecules such as HLA-G on the surface of HRSC, represent paths of immune evasion alternative to the activation of the PD-1/PD-L1 axis, and their prevalence over the latter, either at diagnosis or after therapy with CPIs, might be a key element in primary and secondary resistance to PD-1 directed therapy.

## 6 Immunomodulating therapies targeting the microenvironment

The results from the clinical trials testing CPIs in cHL show how PD-1 targeted therapies are highly effective in this context and put both Pembro and Nivo in the spotlight as main players in the cHL’s treatment algorithm. These results show how the manipulation of immune evasion in the setting of cHL is a very effective therapeutic approach, and, as such, drugs that target some of the other physiopathological mechanisms exploited by HRSC to avoid host recognition could prove to be active and effective, both as standalone and as part of combination therapies.

Favezelimab is a humanised IgG4 monoclonal antibody that targets LAG3 (**Figure 4**), whose activity is currently being explored in combination with Pembro in a phase 1/2 trial (NCT03598608). Notably, cohort 2 of this trial included cHL patients who relapsed after ASCT or were not eligible to undergo the transplant procedure and were refractory to anti-PD-1 therapy. Preliminary results of this trial were updated at the 2022 annual ASH conference. After a median follow-up of 19 months, median PFS was of 10.7 months, while median OS was of 25.7 months and the median duration of response was 19 months, with an ORR of 29% and a CR rate of 9%. Sixty-five percent of patients achieved a response duration of ≥12 months. The 12-month OS and PFS were 91% and 39%, respectively. Notably, 70% of responders had received anti-PD-1 compounds as their most recent line of therapy, suggesting that the efficacy of LAG3-directed therapy might be independent of its combination with PD-1 blockers (107).

Moreover, Camidanlumab-Tesirine (Cami) is a new anti-CD25 antibody-drug conjugate being tested in the setting of R/R cHL (**Figure 4**) in a phase II study whose results have recently been reported in the 2022 EHA conference. The study is being conducted in a large cohort of heavily pretreated patients (n=115; median number of treatments=6), obtaining an ORR of 70.1% and a CRR of 33.3%, respectively, with a median duration of response of 13.7 months. The safety profile for Cami was notable for the development of Guillain-Barré Syndrome in around 7% of patients, an event that needs to be accounted for as the drug is being further tested. Due to the expression of IL-2Rα (CD25) on the surface of both HRSC and Treg cells, the activity of Cami might be attributed, at least partially, to the local depletion of T suppressor cell and the renewal of a competent anti-tumour immune response (108).

## 7 Discussion

Avoidance of the host’s immune system is considered one of the modern hallmarks of cancer (109), and is a key element in the physiopathology of cHL. HRSC achieve immune evasion by building an immune-suppressive ME and through the exploitation of the physiological immune checkpoints. In this context, the PD-1/PD-L1 axis plays a central role, through the overexpression of PD-L1 both on the surface of HRSC and in different non-neoplastic cell types throughout the tumour ME.

After the success of CPIs in the treatment of non-haematological cancers, the efficacy of both Nivo and Pembro has been effectively tested in cHL, achieving impressive results even in canonically harder to treat patients. Interestingly, while in the setting of solid tumours the anticancer activity of PD-1 directed CPIs is mediated by the restoration of killer T-cell function, the usual lack of MCH-I expression in cHL makes this mechanism unlikely, turning our attention to CD4^+^ T-cells and the expression of MHC-II on the surface of HRSC, proven to be a key element in CPI response both by clinical and pathological data. On the other hand, data coming from pathological analysis of biopsies taken only a few days after starting CPI therapy in the context of the NIVAHL trial is suggesting that, at least in the first line setting, it’s not a T-cell response, but rather the loss of pro-survival factors due to depletion in Tregs and macrophages, that is not the driving force for the observed response. Notably, these data contrast quite sharply with the T-cell responses previously described in the relapsed/refractory cHL setting following successful anti-PD-1 therapy (110).

As with many other effective therapeutic tools in the history of cancer, our efforts are now focused on bringing CPIs further up into the cHL’s treatment algorithm, and to improve their efficacy by making them part of combination therapies. The recently published results from the three phase 2 trials mentioned above show how the combination of anti-PD-1 monoclonal antibodies with BV or with chemotherapy schemes such as ICE or GVD as first salvage therapy could improve our cure rates even in primary refractory patients. Moreover, these combinations bring to light the impressive synergistic effect between CPIs and certain chemotherapeutic compounds, associated with the off target immunomodulating effects of agents such as Gemcitabine, Doxorubicin and Vinorelbine and their ability to trigger immunogenic cell death, which makes cancer cells more easily targetable by immune effector cells. The synergy between CPIs and chemotherapy, however, goes both ways, as the exposure to anti-PD-1 seems to render cHL more susceptible to the cytotoxic effects of both chemotherapy and allogenic cellular products in the context of alloHSCT. Further studies are needed to identify the optimal therapeutic approach regarding the specific chemotherapeutic compounds to utilise, the best treatment schedules and the molecular mechanisms underlying these synergisms.

Many ongoing trials are testing varying combinations of CPIs and chemotherapy with a sequential approach or by concurrent administration both in the frontline and relapsed/refractory setting.

As with many other promising agents introduced before them, CPIs are not able to achieve a response in the totality of treated patients, and, especially when used in later lines of therapy and as single agents, responses can be partial and often limited in time, which points to the subsistence of primary and acquired mechanisms of resistance to checkpoint blockade. The existence of alternative strategies of immune escape in the physiopathology of cHL is another fascinating area to explore. While the majority of cHLs seems to largely depend on the aberrant activation of the PD-1/PD-L1 axis to avoid host recognition, the cases that exhibit primary resistance CPIs might be reliant on the subversion of other immune-checkpoints, while in the context of secondary resistance these mechanisms, that may not be prevalent at diagnosis, might take the spotlight away from the PD-1/PD-L1 axis and restore effective immune evasion. Some of these secondary mechanisms are currently being targeted by experimental therapies, which could prove to be effective both following progression while on CPI therapy or in combination with PD-1 blockers. Hopefully, while our experience with these novel agents grows, some clinical, molecular or pathological markers can be identified to properly select the patients that will benefit the most from CPI-based therapies, separating them from the patients that can be effectively cured with a classical chemotherapeutic approach, and from those who wouldn’t benefit from either therapy, delineating a subset of patients that might represent one of the future’s hard-to-solve unmet clinical need.

## Author contributions

AC designed the study, reviewed the literature and wrote the manuscript, FS wrote pathological paragraphs and provided suggestions, MP and LT revised the manuscript and provided suggestions, AV designed the study, reviewed the literature and revised the manuscript. All authors contributed to the article and approved the submitted version.
